# The Distinctive Symptoms*Read before New York State Homœopathic Society.

**Published:** 1884-10

**Authors:** T. L. Brown

**Affiliations:** Binghamton, N. Y.


### THE DISTINCTIVE SYMPTOMS.*

T. L. Brown, M. D., Binghamton, N. Y.

Above all similar considerations, the physician desires to know why he gives one remedy instead of another.

Such knowledge depends upon the one or more known symptoms the remedy produces, differing from those produced by any

---

* Read before New York State Homœopathic Society.

or all the other proven remedies. This symptom must be distinctly understood by the physician or he cannot be satisfied he is giving the right remedy according to the law of "*similia*." If we could always hold in our minds, at each interview with our patient, the great number of symptoms found in our materia medica, we would not need such landmark symptoms to help us determine the remedy.

If each remedy does not produce symptoms not common to other remedies, how could we select a homœopathic remedy suitable to any case?

Experience teaches that one dose of the best attenuated remedy, repeated as often as improvement really ceases, will do the remedial work so much better than flooding the patient with toxical quantities of the medicine. Both schools of medicine begin to more fully understand this fact.

We, therefore, need to know more about the distinguishing symptoms of each remedy we use.

Years of close observation and experience have compelled me to think my best cures, by medicine, have been the most apparent when the distinctive symptoms have guided me in the selection of the curative remedy. Most patients present but few symptoms, and we generally find among those symptoms the ones which best correspond with the indicating symptom of the remedy most needed, and then on further comparison that symptom controls our choice of the remedy.

The abuses of medicine and the bad uses of compound prescriptions will disappear in proportion to the increase of the knowledge gained by the use of one medicine at a time in all prescriptions, and that, too, in the smallest curative doses. The reason we to-day give one remedy and trust it for hours and days, is our knowledge of the guiding symptoms we find so fully corresponding with the symptoms of our patient.

When we give medicines in compound doses and thus fill our patients with a variety of drugs, it will be hard work to tell which has helped or injured the patient the most.

Such patients will commonly recover best on placebos, at least until all drug action ceases to harm them, after which you can the better find the guiding symptoms, leading to the proper choice of remedy.

Our worst patients are those who have been long drugged by both schools into a dose-muddle.

The odd symptom of each patient is the one most likely to direct us sooner to the proper curative remedy—better even than the more common symptoms in the case.

The new and last symptom is the one most likely to aid us in changing to a more suitable remedy.

Disease is not an entity. It is only an organic and functional disorganization of the different portions of the body.

Remedies help reorganize by keeping up the molecular motions necessary to that end. How or why they do it is another question not in my mind to answer. Health is perfect form in body, and timely atomic and molecular motions of all the bodily structures.

Remedies in a toxical form and quantity destroy all human or animal motion, and by their attenuated forms, used at the needed moment, bring back similar health motions controlling organization. The very best drug disorganizers are, when in molecular form, the best organizers when proportionally brought in relative contact.

Organic-molecular motions of the brain-forms, induced by drugs, will increase or diminish mental motions. We therefore very often find the best guiding symptom to be one intimately connected with the mind manifestations. In fact, the mental symptoms are of more importance in the selection of the right remedy than the more common objective symptoms of the other bodily structures.

Our physical and mental conditions, so far as we know, depend upon the correct combination of the elementary, material forms. Hence, when a remedy helps to organize the said elements into bodily organs, it is remedial, and the physical and the mental symptoms of disease disappear.

